# Randomized phase II trial of nimotuzumab plus irinotecan versus irinotecan alone as second-line therapy for patients with advanced gastric cancer

**DOI:** 10.1007/s10120-014-0420-9

**Published:** 2014-09-05

**Authors:** Taroh Satoh, Kyung Hee Lee, Sun Young Rha, Yasutsuna Sasaki, Se Hoon Park, Yoshito Komatsu, Hirofumi Yasui, Tae-You Kim, Kensei Yamaguchi, Nozomu Fuse, Yasuhide Yamada, Takashi Ura, Si-Young Kim, Masaki Munakata, Soh Saitoh, Kazuto Nishio, Satoshi Morita, Eriko Yamamoto, Qingwei Zhang, Jung-mi Kim, Yeul Hong Kim, Yuh Sakata

**Affiliations:** 1 Medical Oncology, Kinki University Faculty of Medicine, Osaka, Japan; 2 Hemato-Oncology, Internal Medicine, Yeungnam University Hospital, Daegu, Republic of Korea; 3 Medical Oncology, Internal Medicine, Yonsei Cancer Center, Yonsei Cancer Research Institute, Yonsei University College of Medicine, Seoul, Republic of Korea; 4 Medical Oncology, Saitama Medical University International Medical Center, Saitama, Japan; 5 Division of Hematology-Oncology, Department of Medicine, Sungkyunkwan University Samsung Medical Center, Seoul, Republic of Korea; 6 Division of Cancer Chemotherapy, Hokkaido University Hospital, Hokkaido, Japan; 7 Division of GI Oncology, Shizuoka Cancer Center, Shizuoka, Japan; 8 Hematology-Oncology, Internal Medicine, Seoul National University Hospital, Seoul, Republic of Korea; 9 Department of Gastroenterology, Saitama Cancer Center, Saitama, Japan; 10 Gastrointestinal Oncology, National Cancer Center Hospital East, Chiba, Japan; 11 Gastrointestinal Oncology, National Cancer Center Hospital, Tokyo, Japan; 12 Department of Clinical Oncology, Aichi Cancer Center Hospital, Aichi, Japan; 13 Department of Medical Oncology and Hematology, Kyung Hee University Hospital, Seoul, Republic of Korea; 14 Internal Medicine, Misawa Municipal Hospital, Aomori, Japan; 15 Medical Oncology and Gastroenterology, Aomori Prefectural Central Hospital, Aomori, Japan; 16 Department of Genome Biology, Kinki University School of Medicine, Osaka, Japan; 17 Department of Biostatics and Epidemiology, Yokohama City University, Kanagawa, Japan; 18 Clinical Development Department II, Daiichi Sankyo Co., Ltd., Tokyo, Japan; 19 Clinical Data and Biostatistics Department, Daiichi Sankyo Co., Ltd., Tokyo, Japan; 20 Medical and Regulatory Affairs Department, Kuhnil Pharm. Co., Ltd., Seoul, Republic of Korea; 21 Department of Internal Medicine, Section of Hemato-Oncology, Korea University Anam Hospital, 126-1 Anam-dong 5ga, Seongbuk-gu, Seoul, 136-705 Republic of Korea

**Keywords:** Nimotuzumab, Anti-EGFR, Irinotecan, Second-line therapy, Advanced gastric cancer

## Abstract

**Background:**

This multicenter, randomized phase II trial was conducted to compare the efficacy and safety of nimotuzumab plus irinotecan (N-IRI) versus irinotecan alone (IRI) in patients with advanced gastric cancer (AGC) showing disease progression after previous 5-fluorouracil-based therapy.

**Methods:**

Irinotecan-naive patients (*n* = 82) received N-IRI (nimotuzumab 400 mg weekly plus irinotecan 150 mg/m^2^ biweekly) or IRI (irinotecan 150 mg/m^2^ biweekly) until disease progression. The primary endpoint was progression-free survival (PFS), and the secondary endpoints were overall survival (OS), response rate (RR), safety, tolerability, and the correlation between efficacy and tumor epidermal growth factor receptor (EGFR) expression.

**Results:**

Of 83 patients, 40 and 43 patients were randomly assigned to the N-IRI and IRI groups, respectively. In the N-IRI/IRI treatment group, median PFS was 73.0/85.0 days (*P* = 0.5668), and median OS and RR at 18 months were 250.5/232.0 days (*P* = 0.9778) and 18.4/10.3 %, respectively. Median PFS and OS in the EGFR 2+/3+ subgroups were 118.5/59.0 and 358.5/229.5 days, respectively. The RR was 33.3/0.0 % in the N-IRI/IRI treatment group. The incidence of grade 3 or higher adverse events was 77.5/64.3 %. No adverse events of grade 3 or higher skin rash or grade 3 or higher infusion-related reaction were reported.

**Conclusions:**

There was no superiority of N-IRI over IRI alone in terms of PFS in 5-fluorouracil-refractory AGC patients. However, N-IRI showed potential improvement in the EGFR 2+/3+ subgroup based on improved RR, PFS, and OS.

## Introduction

Patients with unresectable gastric cancer receiving the best supportive care have poor outcomes, with median survival times ranging from 3 to 5 months [1, 2]. In the metastatic disease setting, palliative chemotherapy improves survival compared with supportive care alone, with combined drug therapy yielding the best results [1–3]. Although there is no universally accepted standard treatment for advanced gastric cancer (AGC), several combination regimens have been used as first-line treatment, including epirubicin–oxaliplatin–capecitabine [4], cisplatin–capecitabine [5], cisplatin-S-1 [6], cisplatin–5-fluorouracil, and docetaxel–cisplatin–5-fluorouracil [7]. However, the median survival has not exceeded 8–13 months [1–7], and second-line treatments need to be established. Irinotecan [8, 9] or paclitaxel monotherapy is commonly used for AGC patients as second-line treatment, especially in Japan and Korea. Because of the limitations of the current therapies, addition of molecular-targeted drugs, particularly to chemotherapies with acceptable toxicities, may improve the outcomes. The ToGA trial demonstrated that the addition of trastuzumab to standard chemotherapy in patients with human EGFR-2 (HER-2)-overexpressing tumors improved overall survival (OS) and progression-free survival (PFS) [10].

Epidermal growth factor receptor (EGFR) is known to be expressed in a variety of tumors [11]. Approximately 30 % of gastric cancers are reported to show EGFR overexpression [12, 13]. EGFR signaling pathways are frequently dysregulated in gastric cancer, thereby serving as attractive therapeutic targets.

Nimotuzumab, a recombinant humanized monoclonal immunoglobulin G_1_ antibody against human EGFR (HER-1), blocks the binding of epidermal growth factor (EGF) and transforming growth factor-α to EGFR. This mechanism regulates antibody-dependent cellular cytotoxicity and complement-dependent cytotoxicity, inhibiting tumor cell growth and angiogenesis and inducing apoptosis [14–17]. In a previous phase I study in Japan, the safety and tolerability of nimotuzumab were investigated up to 400 mg doses weekly [18]. When combined with radiotherapy or chemoradiotherapy, nimotuzumab exerts clinical efficacy against head and neck cancers, gliomas, and non-small cell lung cancer (NSCLC) [17, 19, 20]. Additionally, because of the low frequency of severe dermatological toxicity, nimotuzumab is expected to improve the quality of life.

The present study was an open-label, phase II collaborative study between Japan and Korea. The primary objective was to compare PFS following combined nimotuzumab plus irinotecan therapy (N-IRI) and irinotecan monotherapy (IRI) in patients with unresectable or recurrent gastric cancer refractory to 5-fluorouracil-based therapy.

## Materials and methods

### Patients

Patients in Japan and Korea were enrolled in this multicenter, open-label, randomized phase II trial. Patients with histologically confirmed AGC refractory to previous 5-fluorouracil-based chemotherapy for metastatic disease were eligible. Other major inclusion criteria were as follows: age of 20–75 years; adequate organ function; and Eastern Cooperative Oncology Group performance status (ECOG PS) 0 or 1. Major exclusion criteria were prior exposure to irinotecan or EGFR-directed therapy, and significant comorbidities, such as diarrhea, interstitial pneumonia, or pulmonary fibrosis.

The trial was conducted in accordance with the Declaration of Helsinki. All the patients provided written informed consent. The institutional review boards or ethics committees of all participating centers reviewed and approved the protocol.

### Study treatment

Patients were randomly assigned at a ratio of 1:1 to the N-IRI or IRI group by a computer program on the basis of the resection status of the primary tumor (inoperable advanced/postoperative recurrent) and study site, using the random permuted blocks method. Neither the patients nor the investigators were blinded to the treatment assignment.

Nimotuzumab (400 mg) diluted in normal saline to a total volume of 250 ml was administered once weekly by intravenous infusion over 30 min. Irinotecan (150 mg/m^2^) was administered every 2 weeks. Treatment was continued until disease progression, appearance of unacceptable toxicity, or withdrawal of consent.

### Efficacy and safety assessments

The primary endpoint was PFS following N-IRI versus IRI treatment. PFS was defined as the time from randomization to the day of documentation of progression or death, whichever was earlier. The secondary endpoints were OS, response rate (RR), disease control rate (DCR), safety, and tolerability. Tumor assessment by computed tomography was performed at baseline, and then every 4 weeks for the first 16 weeks, and every 6 weeks thereafter. Evaluation of tumors was performed by an independent Efficacy Evaluation Committee using RECIST 1.0. Adverse events were assessed according to the National Cancer Institute’s Common Terminology Criteria for Adverse Events, version 3.0.

### Exploratory biomarker analysis

EGFR protein expression levels, *EGFR* gene amplification status, *K-ras* mutations, and HER-2 protein expression levels were measured in tissue specimens from tumors obtained from patients who had provided informed consent for exploratory biomarker analysis. The tumor tissues were centrally tested and classified. EGFR expression was analyzed using an immunohistochemistry (IHC) staining kit (EGFR PharmDX; Dako, Copenhagen, Denmark) and classified into four categories (0, 1+, 2+, and 3+), as previously described [21]. The *EGFR* gene copy number was measured by fluorescence in situ hybridization (FISH), as reported previously [22]. For *K-ras* mutation analysis, DNA was extracted from formalin-fixed paraffin-embedded tumor samples. The sequences of *K-ras* codons 12 and 13 and the surrounding region of the gene were analyzed by conventional polymerase chain reaction followed by direct sequencing. The expression status of HER-2 was analyzed using the HercepTest kit (Dako) and classified into four categories (0, 1+, 2+, and 3+).

### Statistical analysis

The reported median PFS in AGC patients treated with irinotecan or paclitaxel as second-line chemotherapy is 2.1–2.6 months [23–25]. For an exploratory study, if the median PFS times for N-IRI and IRI therapy are assumed to be 4.5 and 2.5 months, respectively, then 32 patients per treatment arm would be required to detect a difference with 80 % power at a 10 % significance level using a one-sided log-rank test of the equality of survival curves. Assuming a dropout rate of 20 %, the number of patients per treatment group was set at 40, with a total sample size of at least 80 patients. The median PFS was calculated with the 95 % confidence interval (CI) for both treatment groups. Log-rank tests were performed to evaluate differences in PFS with the significance level set at 10 % (one sided). Primary statistical analysis of the efficacy endpoint was performed 6 months after registration of the last patient for the study.

For subgroup analyses of PFS and OS, the hazard ratio (HR) and 95 % CI within each subgroup were displayed in forest plots. In both treatment groups, the Kaplan–Meier method was used to plot the survival curves and estimate the cumulative incidence from the day of registration to death, as well as the cumulative incidence to disease progression. For evaluation of efficacy, point estimates were calculated for the RR and DCR and compared using the chi-squared test. Efficacy endpoints were analyzed using the full analysis set, safety endpoints were analyzed using the safety analysis set, and pharmacokinetic analyses were performed using the pharmacokinetic analysis set.

## Results

### Patients

**Fig. 1:**
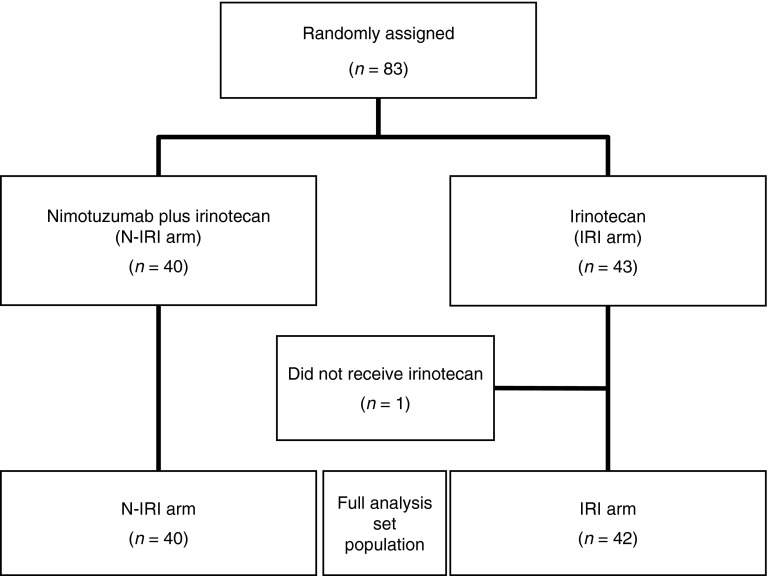
CONSORT diagram. *N-IRI* nimotuzumab plus irinotecan, *IRI* irinotecan alone

A total of 83 patients were randomized from September 2008 to December 2009. Of these patients, 82 were included in the safety and efficacy analysis population (1 patient from the IRI group did not have a target lesion and did not receive irinotecan; Fig. 1). At the 18-month follow-up after registration of the last patient for the study, the median nimotuzumab and irinotecan exposure in the N-IRI group was 71.5 days (range, 8–947) and 60.5 days (range, 1–268), respectively, and the median irinotecan exposure in the IRI group was 57.0 days (range, 1–953). The median follow-up period was 242.5 days (range, 22–955). Patient demographics, including the UGT1A1 subtype, were well matched between the two treatment groups (Table 1).

**Table 1:** Baseline characteristics of the patients

Characteristics	N-IRI arm (*n* = 40)		IRI arm (*n* = 42)		Total (*n* = 82)	
	*n*	%	*n*	%	*n*	%
Age (years)						
Median	60.0		63.5		61.5	
Range	27–75		32–75		27–75	
Sex						
Male	33	82.5	33	78.6	66	80.5
Female	7	17.5	9	21.4	16	19.5
ECOG performance status						
0	19	47.5	17	40.5	36	43.9
1	21	52.5	25	59.5	46	56.1
Body weight (kg)						
Median	56.3		54.2		56.0	
Range	42.0–81.4		37.5–107.0		37.5–107.0	
Resection status of the primary tumor						
Inoperable advanced	22	55.0	23	54.8	45	54.9
Postoperative recurrent	18	45.0	19	45.2	37	45.1
Histological diagnosis, *n*						
Well/moderately differentiated adenocarcinoma	15	37.5	19	45.2	34	41.5
Poorly differentiated adenocarcinoma	21	52.5	17	40.5	38	46.3
Others	4	10.0	6	14.3	10	12.2
Primary tumor site						
Absent	18	45.0	16	38.1	34	41.5
Present	22	55.0	26	61.9	48	58.5
Gastroesophageal junction	4	18.2	1	3.8	5	10.4
Gastric region	18	81.8	25	96.2	43	89.6
Metastatic focus site						
No	1	2.5	0	0.0	1	1.2
Yes	39	97.5	42	100.0	81	98.8
Lymph node	25	64.1	25	59.5	50	61.7
Liver	13	33.3	19	45.2	32	39.5
Lung	3	7.7	6	14.3	9	11.1
Other	19	48.7	18	42.9	37	45.7
UGT1A1 gene polymorphism						
*1/*1, *1/*6, *1/*28	38	95.0	39	92.9	77	93.9
*6/*6, *28/*28, *6/*28	2	5.0	3	7.1	5	6.1

*N-IRI* nimotuzumab plus irinotecan, *IRI* irinotecan alone, *n* number of patients, *ECOG* Eastern Cooperative Oncology Group

Of the 83 patients, 48 patients had provided informed consent for exploratory biomarker analysis and submitted tumor samples. The EGFR protein expression level was detected in the assessable tumor tissues of 47 patients (57.3 % of the full analysis set population) (Table 2).

**Table 2:** EGFR and HER-2 protein expression levels identified by immunohistochemistry

	EGFR							
	0		1+		2+, 3+		Total	
	*n*	%	*n*	%	*n*	%	*n*	%
HER2								
0	15	31.3	8	16.7	8	16.7	31	64.6
1+	2	4.2	2	4.2	5	10.4	9	18.8
2+, 3+	4	8.3	2	4.2	1	2.1	7	14.6
Total	21	43.8	12	25.0	14	29.2	47^a^	97.9

*EGFR* epidermal growth factor receptor, *n* number of patients, *HER2* human EGFR-2

^a^One sample was “not detected”

### Efficacy

**Fig. 2:**
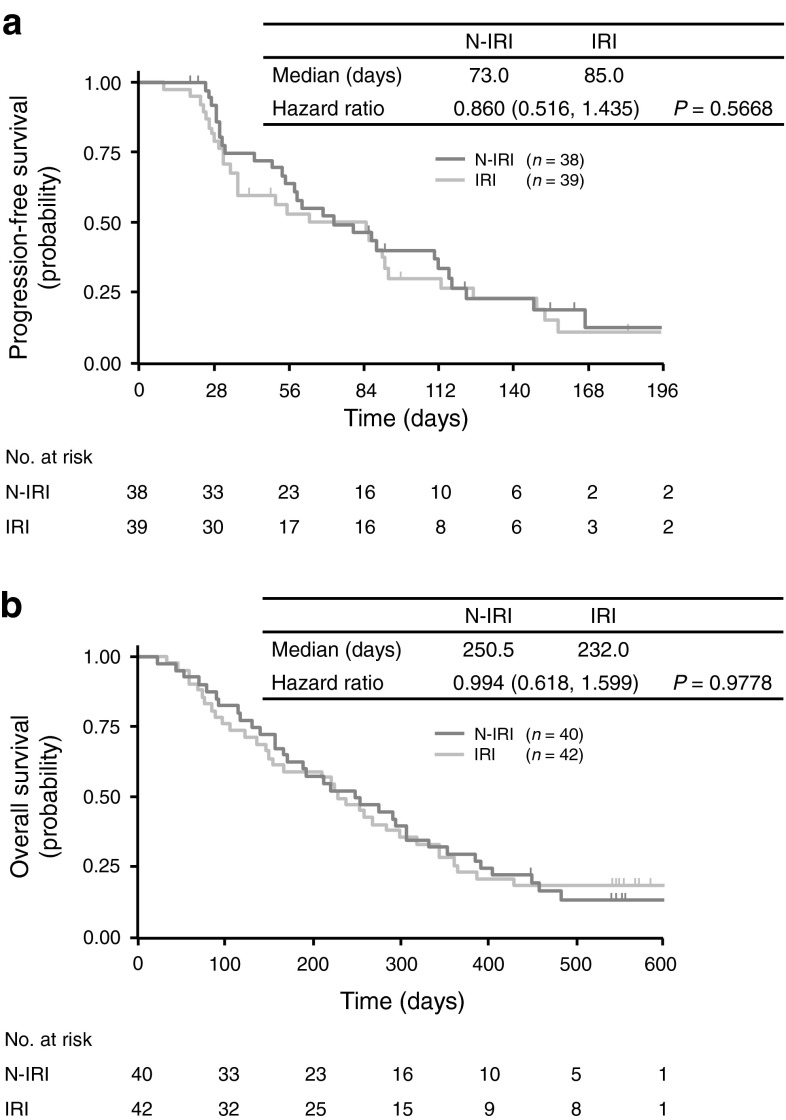
Kaplan–Meier estimates of progression-free survival (**a**) and overall survival (**b**). *N-IRI* nimotuzumab plus irinotecan, *IRI* irinotecan alone

A total of 77 patients (*n* = 38 in the N-IRI group and *n* = 39 in the IRI group) were evaluable for radiologic tumor responses by an Independent Efficacy Evaluation Committee. PFS evaluated at 6 months after registration of the last patient was not significantly different between the treatment groups [median (95 % CI), 73.0 (55.0–112.0) days in the N-IRI group vs. 85.0 (37.0–93.0) days in the IRI group; HR (95 % CI), 0.860 (0.516–1.435), *P* = 0.5668] (Fig. 2).

By 18 months after registration of the last patient, 34 patients from each group had died and the 18-month OS was not significantly different between the treatment groups [median (95 % CI), 250.5 (171.0–306.0) days in the N-IRI group vs. 232.0 (148.0–319.0) days in the IRI group; HR (95 % CI), 0.994 (0.618–1.599), *P* = 0.9778]. There was no significant difference in RR or DCR at the 18-month follow-up between the treatment groups (RR, 18.4 % in the N-IRI group vs. 10.3 % in the IRI group, *P* = 0.3060; DCR, 47.4 % in the N-IRI group vs. 46.2 % in the IRI group, *P* = 0.9150).

**Fig. 3:**
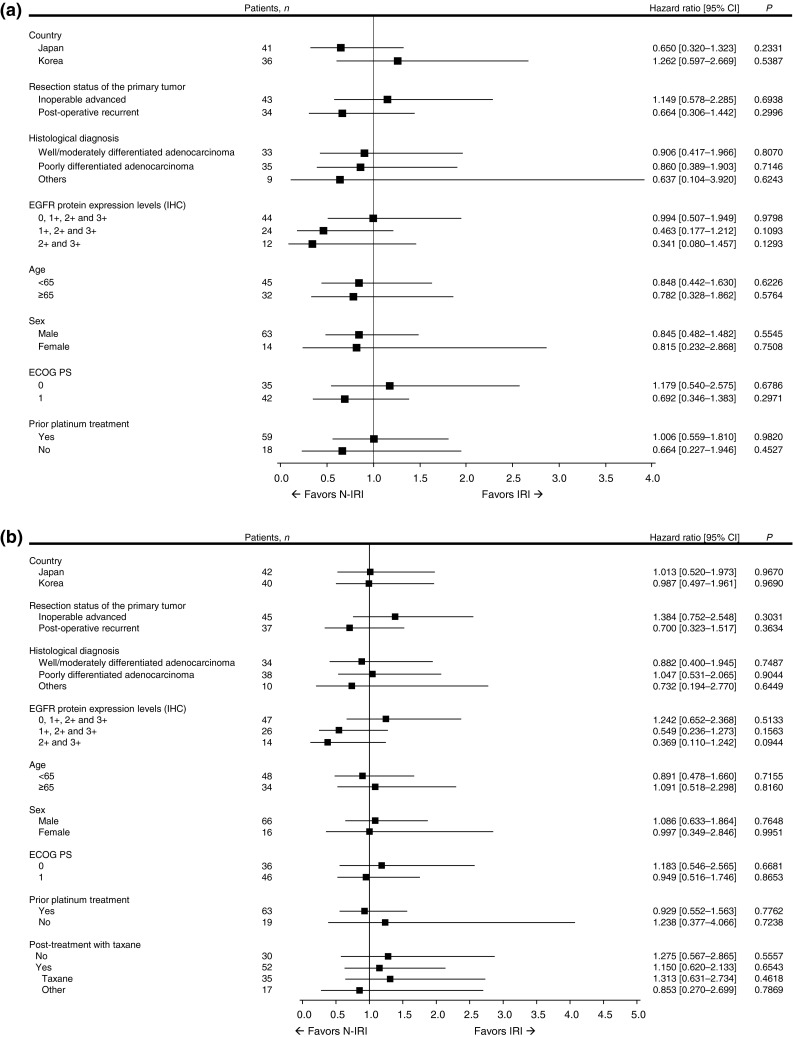
Subset forest plots for progression-free survival (**a**) and overall survival (**b**). *N-IRI* nimotuzumab plus irinotecan, *IRI* irinotecan alone, *EGFR* epidermal growth factor receptor, *IHC* immunohistochemistry, *ECOG* Eastern Cooperative Oncology Group, *PS* performance status

PFS and OS in the various subgroups analyzed were not significantly different between the treatment groups (Fig. 3). However, the HR (IRI/N-IRI) in the EGFR 2+/3+ subgroup was lower than that in the entire treatment group. First, the median PFS (95 % CI) was 118.5 (87.0–not estimated) days for six patients in the N-IRI group vs. 59.0 (24.0–113.0) days for six patients in the IRI group [HR (95 % CI), 0.341 (0.080–1.457), *P* = 0.1293] (Fig. 3). Second, the median OS (95 % CI) was 358.5 (274.0–458.0) days for six patients in the N-IRI group vs. 229.5 (58.0–387.0) days for eight patients in the IRI group [HR (95 % CI), 0.369 (0.110–1.242), *P* = 0.0944] (Fig. 3). Furthermore, at the 18-month follow-up, the RR in the EGFR 2+/3+ subgroup was 33.3 % for six patients in the N-IRI group vs. 0.0 % for six patients in the IRI group, and the DCR in the corresponding groups was 83.3 % and 33.3 %, respectively.

### Pharmacokinetics

Pharmacokinetic analysis was conducted using data collected from 11 patients (*n* = 6 from the N-IRI group and *n* = 5 from the IRI group). The pharmacokinetic parameters of nimotuzumab were similar to those reported from a previous phase I study of nimotuzumab in Japanese patients with solid tumors [18].

### Safety

Adverse events were reported in all the patients. Table 3 shows the incidence, by treatment group, of major adverse events occurring at a frequency of ≥15 % in at least one group. The most common adverse events (≥50 % in at least one group) were neutropenia, diarrhea, nausea, alopecia, decreased appetite, fatigue, and leukopenia. A rash occurred in 25.0 % (10/40) and 4.8 % (2/42) of patients in the N-IRI and IRI groups, respectively. There were no cases with severe (≥grade 3) skin toxicity, including severe rash. Grade 3 or higher adverse events were encountered in 77.5 % of patients in the N-IRI group and 64.3 % of patients in the IRI group. The most common grade 3 or higher adverse events (≥10 % in at least one group) were neutropenia, nausea, leukopenia, anemia, pneumonia, and decreased hemoglobin. The two pneumonia-related deaths in the N-IRI group were considered to be causally related to the study drug. All patients with pneumonia were evaluated by an Independent Data Monitoring Committee to detect pneumonitis. However, no cases of pneumonitis were identified.

**Table 3:** Adverse events occurring at an incidence of ≥ 15 % in each treatment arm

Adverse event	N-IRI (*n* = 40)				IRI (*n* = 42)			
	All grades		≥Grade 3		All grades		≥Grade 3	
	*n*	%	*n*	%	*n*	%	*n*	%
Infections and infestations								
Pneumonia	8	20.0	4	10.0	1	2.4	0	0.0
Blood and lymphatic system disorders								
Anemia	7	17.5	5	12.5	4	9.5	3	7.1
Leukopenia	20	50.0	6	15.0	15	35.7	4	9.5
Lymphopenia	7	17.5	3	7.5	4	9.5	0	0.0
Neutropenia	29	72.5	18	45.0	23	54.8	16	38.1
Thrombocytopenia	1	2.5	0	0.0	7	16.7	3	7.1
Metabolism and nutrition disorders								
Hypoalbuminemia	7	17.5	0	0.0	5	11.9	1	2.4
Decreased appetite	22	55.0	3	7.5	26	61.9	3	7.1
Gastrointestinal disorders								
Abdominal pain	14	35.0	0	0.0	14	33.3	3	7.1
Constipation	12	30.0	0	0.0	12	28.6	0	0.0
Diarrhea	25	62.5	3	7.5	25	59.5	2	4.8
Nausea	25	62.5	6	15.0	25	59.5	4	9.5
Stomatitis	6	15.0	0	0.0	5	11.9	0	0.0
Vomiting	17	42.5	3	7.5	13	31.0	2	4.8
Skin and subcutaneous tissue disorders								
Alopecia	23	57.5	0	0.0	15	35.7	0	0.0
Rash	10	25.0	0	0.0	2	4.8	0	0.0
General disorders and administration site conditions								
Asthenia	7	17.5	1	2.5	9	21.4	1	2.4
Fatigue	21	52.5	3	7.5	15	35.7	3	7.1
Pyrexia	8	20.0	0	0.0	13	31.0	0	0.0
Investigations								
Alanine aminotransferase increased	8	20.0	1	2.5	6	14.3	1	2.4
Aspartate aminotransferase increased	7	17.5	1	2.5	7	16.7	1	2.4
Hemoglobin decreased	11	27.5	4	10.0	13	31.0	6	14.3
Weight decreased	12	30.0	2	5.0	8	19.0	0	0.0

Number of patients, incidence of adverse events, and incidence of grades 3–5 adverse events

*N*-*IRI* nimotuzumab plus irinotecan, *IRI* irinotecan alone, *n* number of patients

The incidence of adverse events resulting in discontinuation of irinotecan was 15.0 % (6/40) in the N-IRI group and 16.7 % (7/42) in the IRI group, with no significant difference between the two groups. The incidence of adverse events resulting in discontinuation of nimotuzumab was 7.5 % (3/40) in the N-IRI treatment group.

Adverse events were reported for all patients in the EGFR 2+/3+ subgroup, and no significant difference was found in the frequency of adverse events between the IRI and N-IRI groups in the EGFR 2+/3+ subgroup analysis. The incidence of adverse events in the EGFR 2+/3+ subgroup was similar to that in all randomized patients. In the EGFR 2+/3+ subgroup, rash of grade 1 or 2 occurred in 50.0 % (3/6) and 0.0 % (0/8) of patients in the N-IRI and IRI groups, respectively.

## Discussion

The primary endpoint of prolonged PFS was not achieved in this study, suggesting no significant benefit of N-IRI in non-biologically selected patients with AGC. This result is suggested by recent studies that evaluated the efficacy of anti-EGFR antibody administration to AGC patients who were not biologically selected. In two prospective randomized phase III studies (EXPAND, REAL-3) of cetuximab and panitumumab conducted in AGC patients, the primary endpoint could not be achieved [26, 27]. These negative results emphasize the need to identify the biological target before starting a large phase III study.

In a preclinical study, nimotuzumab showed marked antiproliferative, proapoptotic, and antiangiogenic effects against tumors showing EGFR overexpression [14–16]. We previously demonstrated that the effects of nimotuzumab on human NSCLC cell lines were highly dependent on EGFR status [28]. Nimotuzumab inhibited EGFR phosphorylation in cancer cells with high/moderate surface expression of EGFR, but not in those with low surface EGFR expression. Immunoblot analysis showed inhibition of EGFR phosphorylation in H292 and Ma-1 cells expressing high and moderate levels of EGFR on the cell surface, but not in H460, H1299, and H1975 cells showing a low level of surface EGFR expression [28].

In a clinical study of head and neck cancer to assess the efficacy of nimotuzumab in combination with radiotherapy, a controlled, double-blind, randomized clinical trial was conducted. For EGFR-positive patients, a significant survival improvement was detected for nimotuzumab-treated patients (OS, 16.5 months) compared with the control group (OS, 7.2 months) [29].

Nimotuzumab is a humanized IgG1 antibody-directed agent, meaning that EGFR should be considered as the first candidate for its biological target. In this study, subset analysis showed a median PFS of 118.5 days in the EGFR 2+/3+ subgroup of the N-IRI group and 59.0 days in the corresponding subgroup of the IRI group; the RR was 33.3 % and 0.0 %, respectively. Furthermore, there was no significant difference in the frequency and seriousness of adverse events between the IRI and N-IRI groups in the subset of EGFR 2+/3+ subgroup analysis. Submission of tissue samples was not mandatory, and EGFR protein expression was only detected for 57.3 % of the full analysis set population. Therefore, the subset analysis based on the EGFR status could not yield any conclusive results. However, the results seem to imply that nimotuzumab can improve PFS and OS in AGC patients with high EGFR expression levels (2+/3+) when administered in combination with irinotecan.

In our study, the further exploratory biomarker of *K-ras* mutations was measured in 48 patients, and only 2 patients were found to harbor *K-ras* mutations. The *EGFR* gene copy number was measured in 46 patients, and 1 patient was detected with gene amplification. These results were consistent with previous reports [30, 31]. The roles of *K-ras* mutations and *EGFR* gene amplification were not clear in this study.

Recently, the ToGA study showed that the HER-2-targeting monoclonal antibody trastuzumab improved OS in AGC patients with HER-2 protein overexpression by IHC or gene amplification by FISH [10]. We also investigated the HER-2 expression levels by IHC and found that 14.6 % (7/48) of patients showed HER-2 2+/3+ expression and 29.2 % (14/48) of patients showed EGFR 2+/3+ expression. Only 2.1 % (1/48) of patients showed 2+/3+ expression of both EGFR and HER-2, suggesting there is little overlap between EGFR and HER-2 overexpression in gastric cancer [13]. Currently, targeted therapy for gastric cancer is limited to patients with HER-2 overexpression. However, in future, patients with gastric cancer showing EGFR overexpression might benefit from treatment with nimotuzumab.

In the present study, rash occurred in ten patients (25.0 %) in the N-IRI group, which represents a lower frequency than that reported for patients receiving other anti-EGFR monoclonal antibodies, such as cetuximab or panitumumab [26, 27]. Furthermore, there were no cases of severe (≥grade 3) skin toxicity in either treatment group. The frequency and severity of skin toxicity associated with nimotuzumab appears to be lower than that associated with other anti-EGFR antibodies. The safety profile of nimotuzumab could be expected to maintain good quality of life as well as compliance and shows potential for combination of nimotuzumab with irinotecan. The median relative dose intensity of irinotecan and nimotuzumab was 94.94 % and 96.55 %, respectively, in the N-IRI group. In the study of REAL-3, compliance with the baseline chemotherapy was decreased because of some severe toxicities, and the combination of a triple-chemotherapy regimen with panitumumab appears to be difficult to deliver [27].

The mechanism underlying this lower frequency of skin toxicity of nimotuzumab compared with that of other known anti-EGFR antibodies has been investigated in several recent studies [14–17, 19, 20]. These studies suggested that a low incidence of skin toxicity may be associated with the following: (1) the intermediate affinity (*K*_d_ = 10^−8^ M) of nimotuzumab, which is at least one order of magnitude lower than that of cetuximab or panitumumab; and (2) the different binding profile of nimotuzumab, which requires bivalent binding for stable attachment to the cellular surface compared with that of cetuximab, which requires only monovalent binding [15, 16]. This finding implies that nimotuzumab binding to EGFR occurs only when the surface EGFR density is sufficiently high to allow bivalent binding. Tumor cells overexpressing EGFR are common, allowing for selective binding of nimotuzumab.

In conclusion, although the primary endpoint of prolonged PFS was not met in our study, subset analysis showed that the N-IRI regimen may have potential to improve PFS and OS in EGFR 2+/3+ patients. An open-label, randomized phase III trial comparing N-IRI and IRI in EGFR 2+/3+ AGC patients is currently ongoing.
